# A modified elliptical formula to estimate kidney collagen content in a model of chronic kidney disease

**DOI:** 10.1371/journal.pone.0190815

**Published:** 2018-01-19

**Authors:** Jake A. Nieto, Janice Zhu, Bin Duan, Jingsong Li, Ping Zhou, Latha Paka, Michael A. Yamin, Itzhak D. Goldberg, Prakash Narayan

**Affiliations:** Department of Preclinical Research, Angion Biomedica Corp., Uniondale, New York, United States of America; The University of Tokyo, JAPAN

## Abstract

The extent of scarring or renal interstitial collagen deposition in chronic kidney disease (CKD) can only be ascertained by highly invasive, painful and sometimes risky, tissue biopsy. Interestingly, while CKD-related abnormalities in kidney size can often be visualized using ultrasound, not only does the ellipsoid formula used today underestimate true renal size, but the calculated renal size does not inform tubulointerstitial collagen content. We used coronal kidney sections from healthy mice and mice with kidney disease to develop a new formula for estimating renal parenchymal area. While treating the kidney as an ellipse with the major axis (a) the polar distance, this technique involves extending the minor axis (b) into the renal pelvis to obtain a new minor axis, b_e_. The calculated renal parenchymal area is remarkably similar to the true or measured area. Biochemically determined kidney collagen content revealed a strong and positive correlation with the calculated renal parenchymal area. Picrosirius red staining for tubulointerstitial collagen also correlated with calculated renal parenchymal area. The extent of renal scarring, i.e. kidney interstitial collagen content, can now be computed by making just two axial measurements which can easily be accomplished via noninvasive imaging of this organ.

## Introduction

Given the prevalence of diabetes, hypertension and Metabolic Syndrome, chronic kidney disease (CKD) is reaching epidemic proportions across the world [1,2]. Characterized by scarring or accumulation of fibrillar collagen within the renal interstitium, CKD is associated with a progressive decline in renal function. However, existing disease is often diagnosed late because clinically meaningful changes in renal function occur long after substantial and irreversible scar formation [3,4]. Further compounding both disease diagnosis and prognosis is the fact that highly invasive renal biopsy remains the mainstay for determining the extent of renal scarring [5]. Interestingly, fibrosis-related abnormalities in kidney dimension can be visualized by noninvasive sonography [6–8]. Renal length or major axis, renal width or minor axis and renal thickness measurements can be incorporated into an ellipsoid formula to yield kidney size [9–11]. Unfortunately, renal dimension does not inform dimension with the amount of tissue interstitial collagen. Second, the standard ellipsoid formula underestimates true kidney size confounding any inferences of tissue collagen content [9–11].

In the present study, we used kidneys from healthy mice and mice with CKD to develop a modified elliptical formula that better represents true renal parenchymal area. We then formulated a relationship between calculated renal parenchymal area and total kidney collagen.

## Methods

### Animal model

The study protocol, designed to induce renal fibrosis in mice, was submitted to and approved by the Angion Biomedica Corp. Institutional Animal Care and Use and Committee. Animals were allowed to acclimatize for a minimum of 5 days prior to use and had free access to water and standard rodent chow. Adult male CD-1 mice (~30–35 g) were anesthetized with ketamine (25 mg/kg, ip) and xylazine (5 mg/kg, ip) and placed on a heating pad table to maintain ~37.5°C core body temperature. A midline laparotomy was made and the right kidney removed was removed. Extended release buprenorphine (0.65 mg/kg, sc) was administered prior to returning animals to their cages. One week later, animals were placed on 1% NaCl (drinking water) and deoxycorticosterone acetate (DOCA, 1 mg/kg, sc) injected twice weekly for the first 3 weeks [12,13]. Eight weeks after nephrectomy, animals were sacrificed and the left kidney retrieved. Age-matched, surgery-and DOCA-naive animals on regular drinking water were used as the baseline control. Left kidneys from these animals were retrieved at sacrifice. Kidneys were weighed and sliced coronally under a dissecting microscope (4X). One half of the kidney was placed in 10% formalin for subsequent sectioning (coronal sections 5 μm apart) and staining with hematoxylin & eosin (H&E). The other half of the kidney was weighed and submitted to analysis for collagen content.

### Kidney collagen content

Total kidney collagen content was determined biochemically by measuring tissue hydroxyproline as described previously [14]. Total kidney hydroxyproline values were converted to total kidney collagen (μg/kidney) content [14]. The pattern of collagen deposition, typically tubulointerstitial, was visualized using Picrosirius red staining [14] semi-quantified by a blinded observer using the Bioquant Image Analysis

### Renal parenchymal area

H&E-stained renal sections were photographed (Nikon) and analyzed using NIS-Elements D 3.1 software by an observer blinded to the collagen content of that kidney. Images were superimposed on a precalibrated grid of 1 mm resolution and the major (a), minor (b) and the extended minor (b_e_) axes measured. True renal parenchymal area (mm^2^) was measured using the “area measurement” tool available in the software and also calculated from Eqs 1 and 2.

### Data analysis

Total kidney collagen content and the corresponding renal parenchymal area measurement were obtained from a total of 280 kidneys, 10 from the healthy cohort and 18 from the diseased cohort. The pattern of collagen deposition was visualized in 6 (healthy+diseased) kidneys and Picrosirius red-stained area quantified as a percentage of the total field area. Microsoft Excel 2010 curve fitting software was used to generate all scatterplots. Since a linear relation was observed between the 2 variables in each of the scatterplots, both Pearson product moment (r) and Spearman’s rho (*r*_*s*_) were calculated from the trend line. To determine whether the relationship between the 2 variables was significant, r or *r*_*s*_ and the sample size (n = 30) were entered into an online calculator [15]. A p <0.05 was considered to be statistically significant.

## Results

**Fig 1:**
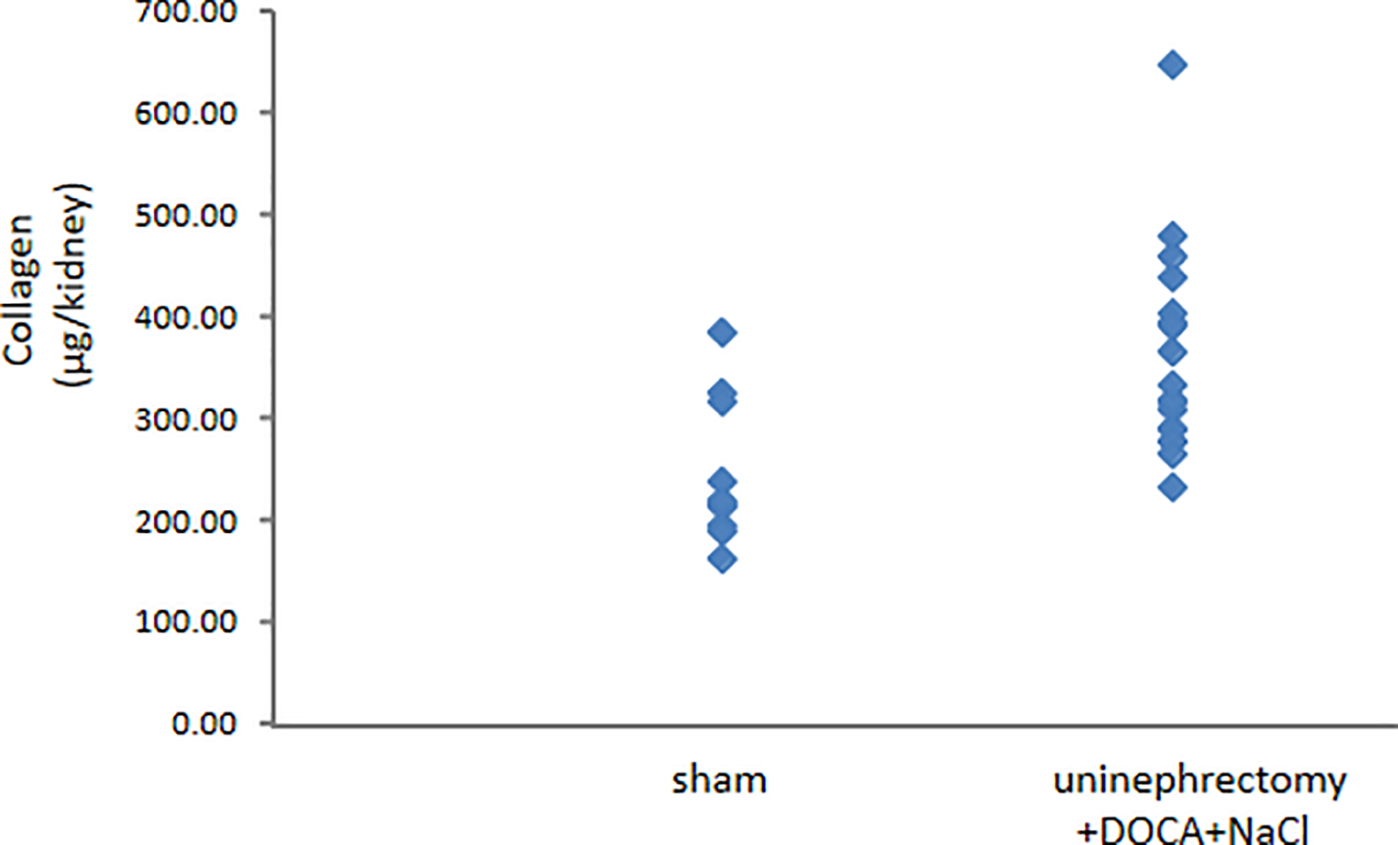
Kidney collagen content in healthy and diseased mice. Total kidney collagen content for each healthy mouse and each uninephrectomized mouse administered DOCA+NaCl is shown. In general, mice from the latter cohort exhibited higher kidney collagen content albeit there is a clear evidence of a distribution of collagen content in kidneys of mice within each cohort.

Total collagen content in individual kidneys spanned a 4-fold dynamic range, from 163 μg/kidney to 647 μg/kidney (Fig 1). Compared to the sham cohort, kidneys from the uninephrectomy+DOCA+NaCl cohort exhibited on average a 46% increase in collagen content (247±22 μg/kidney vs. 361±24 μg/kidney, respectively; p<0.01).

**Fig 2:**
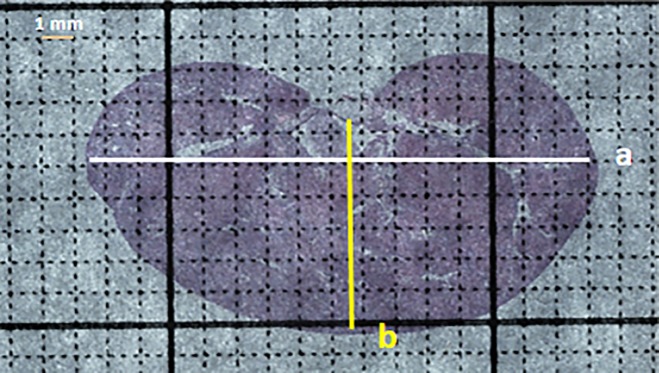
Renal parenchyma area. An H&E-stained coronal section (4X) from the left kidney of a uninephrectomized mouse administered DOCA and NaCl. The section has been superimposed on a 1 mm^2^ grid. The white bar represents renal length or the major axis (a) whereas the yellow bar represents renal width or the minor axis (b). The renal parenchymal area can be measured using a precalibrated measuring tool or calculated from a and b.

**Fig 3:**
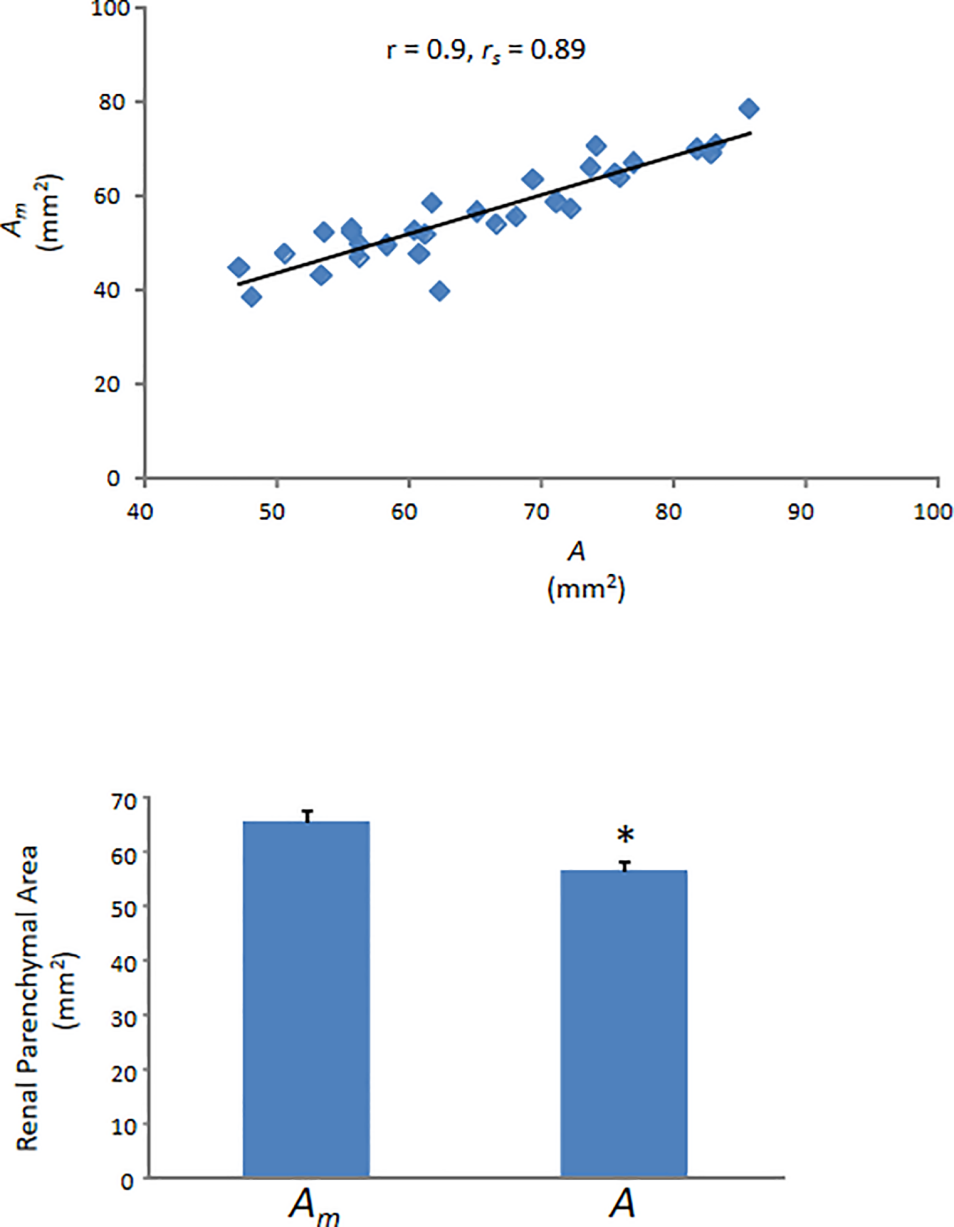
Measured (*A*_*m*_) vs. Calculated (*A*) renal parenchymal areas. (Top) A scatter plot of *A*_*m*_, the measured renal parenchymal area vs. *A*, the renal parenchymal area calculated by entering the axial dimensions, length (a) and width (b), into the elliptical formula. The correlation was significant (p < 0.01). (Bottom) The calculated parenchymal area from 30 kidneys is only 86% of the measured parenchymal area. This difference is significant (*. p < 0.01).

An H&E-stained renal coronal section from a uninephrectomized mouse administered DOCA+NaCl with the major (a) and minor (b) axes delineated is shown (Fig 2). The equation for the area of an ellipse, viz,

A=(π*a*b)/4 (1)

was used to calculate the renal parenchyma area (*A*) and correlate it with the measured parenchymal area (*A*_*m*_). There is a very high correlation (Fig 3) between these two variables (r = 0.9, p < 0.01; *r*_*s*_ = 0.89, p < 0.01) [16]. Nevertheless, consistent with the published literature [9–11], use of the standard elliptical formula underestimates true renal dimension as *A* was only 86 ± 1% of *A*_*m*_ (p < 0.01; Fig 3).

**Fig 4:**
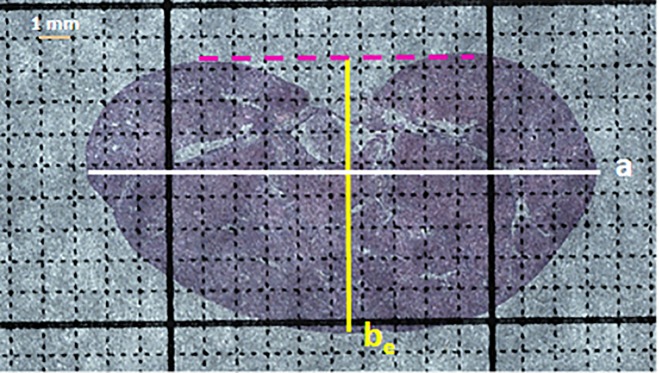
Renal parenchyma area. An H&E-stained coronal section (4X) from the left kidney of a uninephrectomized mouse administered DOCA and NaCl. The section has been superimposed on a 1 mm^2^ grid. The white bar represents renal length or the major axis (a) whereas the yellow bar represents the extended minor axis (b_e_), which has been extended into the renal pelvis until it intersects the pink dashed bar. The renal parenchymal area can be measured using a precalibrated measuring tool or calculated from a, and b_e_.

To obtain greater fidelity toward *A*_*m*_, the minor axis was extended (b_e_) into the renal pelvis (Fig 4). A modified elliptical area equation, viz.

**Fig 5:**
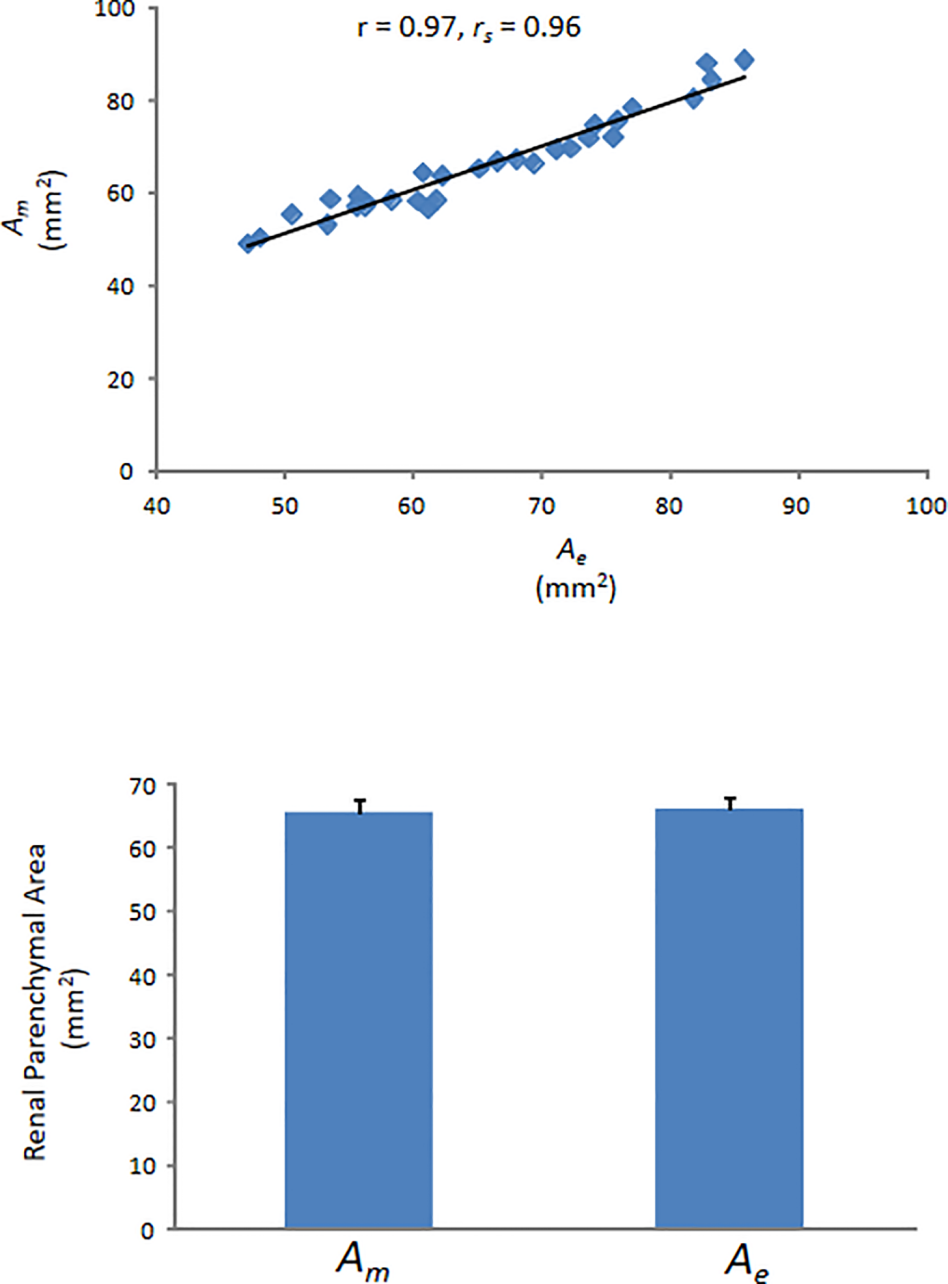
Measured (*A*_*m*_) vs. Calculated (*A*_*e*_) renal parenchymal areas. (Top) A scatter plot of *A*_*m*_, the measured renal parenchymal area vs. *A*_*e*_, the renal parenchymal area calculated by entering the axial dimensions of length (a) and modified width (b_e_) into the elliptical formula. The correlation was significant (p < 0.01). (Bottom) The calculated average parenchymal area from 30 kidneys is not different from the measured areas.

Ae=(π*a*be)/4 (2)

was used to recalculate renal parenchymal areas (*A*_*e*_) and correlate it with *A*_*m*_. Use of an extended minor axis in the elliptical equation returned calculated areas that correlated (Fig 5) very highly with the measured areas (r = 0.97, p < 0.01; *r*_*s*_ = 0.96, p < 0.01) [16]. Importantly, for the sample set, *A*_*e*_ was 100.9 ± 0.7% of *A*_*m*_ (p not significant; Fig 5).

**Fig 6:**
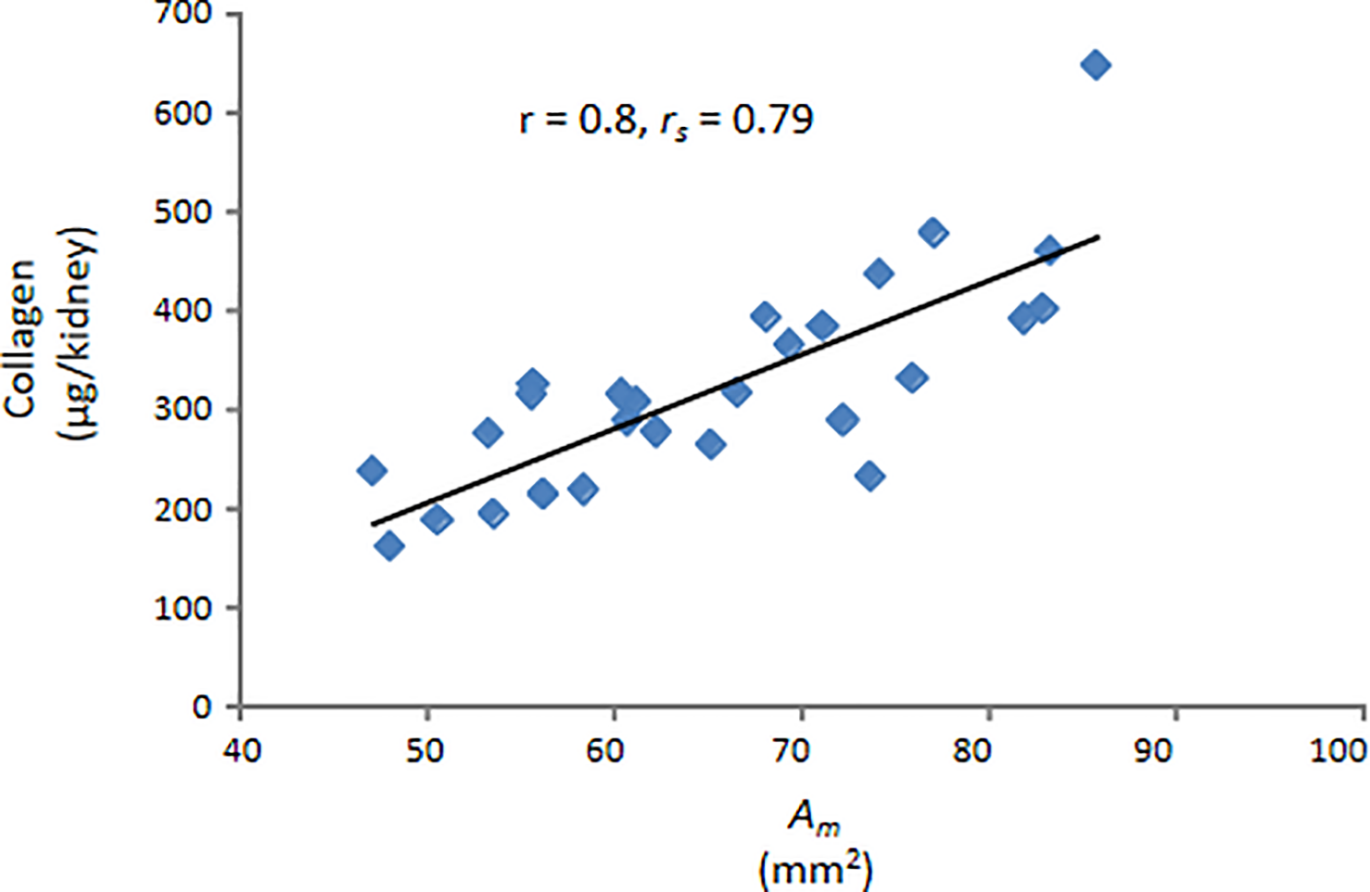
Kidney collagen vs. measured renal parenchymal area. Collagen content from healthy and diseased kidneys was correlated with the corresponding measured renal parenchymal areas (*A*_*m*_). The correlation was significant.

Total collagen content of each kidney from the sham or diseased groups was plotted against that kidneys measured parenchymal area i.e. *A*_*m*_. A high correlation (Fig 6) was observed between kidney collagen and *A*_*m*_. (r = 0.8, p < 0.01, *r*_*s*_ = 0.79, p < 0.01) [16].

**Fig 7:**
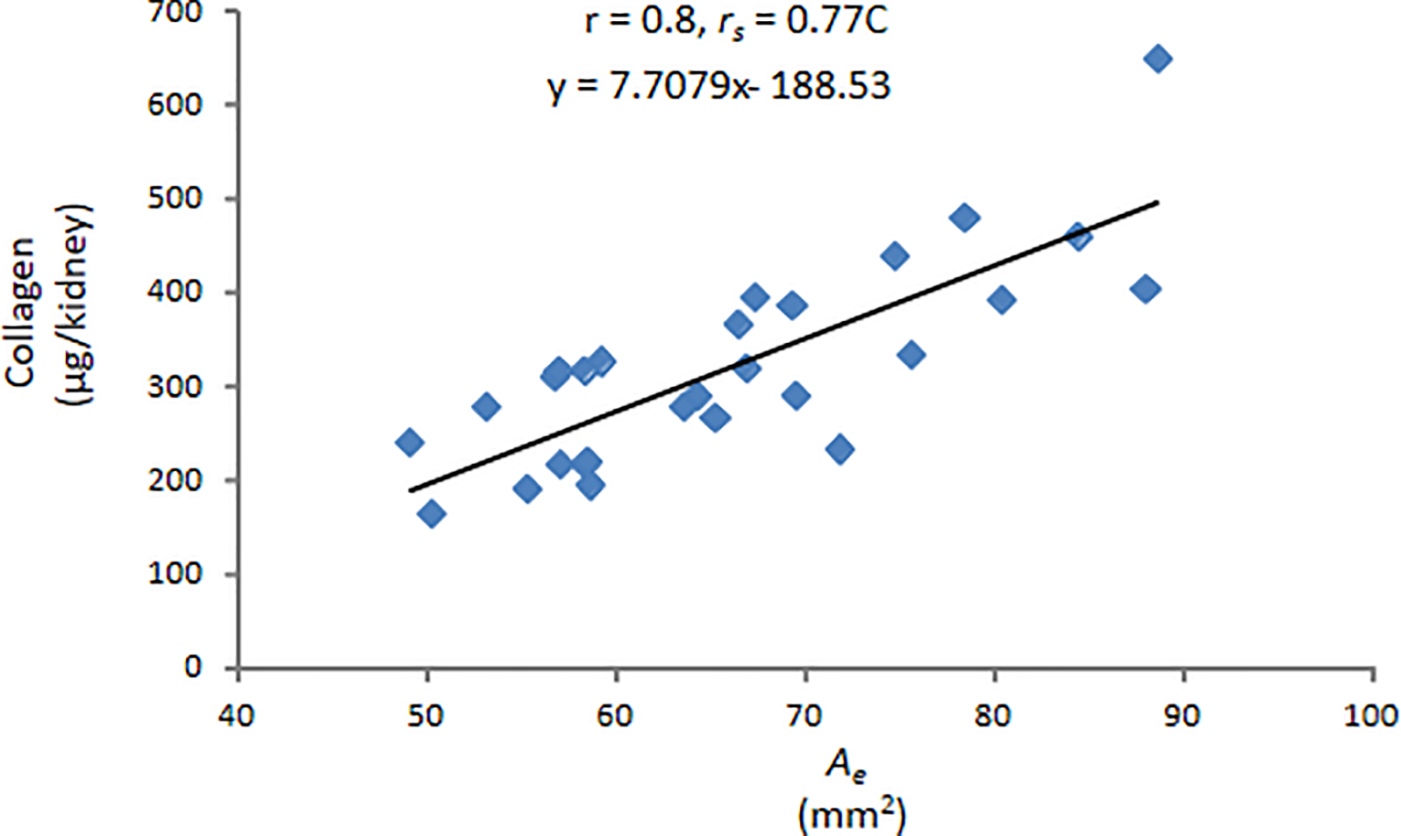
Kidney collagen vs. calculated renal parenchymal area. Collagen content from healthy and diseased kidneys was correlated with the corresponding renal parenchymal areas (*A*_*e*_) calculated using an elliptical equation with an extended minor axis (b_e_). The correlation was significant. The equation inset describes the relation between kidney collagen content and *A*_*e*_.

Plotting kidney total collagen content vs. *A*_*e*_ also yielded a high correlation (Fig 7) with r = 0.8, p < 0.01, *r*_*s*_ = 0.77, p < 0.01 [17]. In fact, the correlation with kidney total collagen for *A*_*m*_ or *A*_*e*_ was very similar, once again suggesting that the calculated area, *A*_*e*_, can be substituted for the true or measured area, *A*_*m*_.

The relation between kidney collagen content and renal parenchymal area can be described by the following formula

y=[7.7*x−188.5] (3)

Since,

x=Ae=(π*a*be/4) (4)

Collagen(μg/kidney)=[7.7*(π*a*be/4)−188.5] (5)

Formalin-fixed sections from Picrosirius red staining of a subset of kidneys was performed as an additional marker of fibrosis and equally importantly, as an aid to visualize the pattern of collagen deposition vs. the renal parenchymal area.

**Fig 8:**
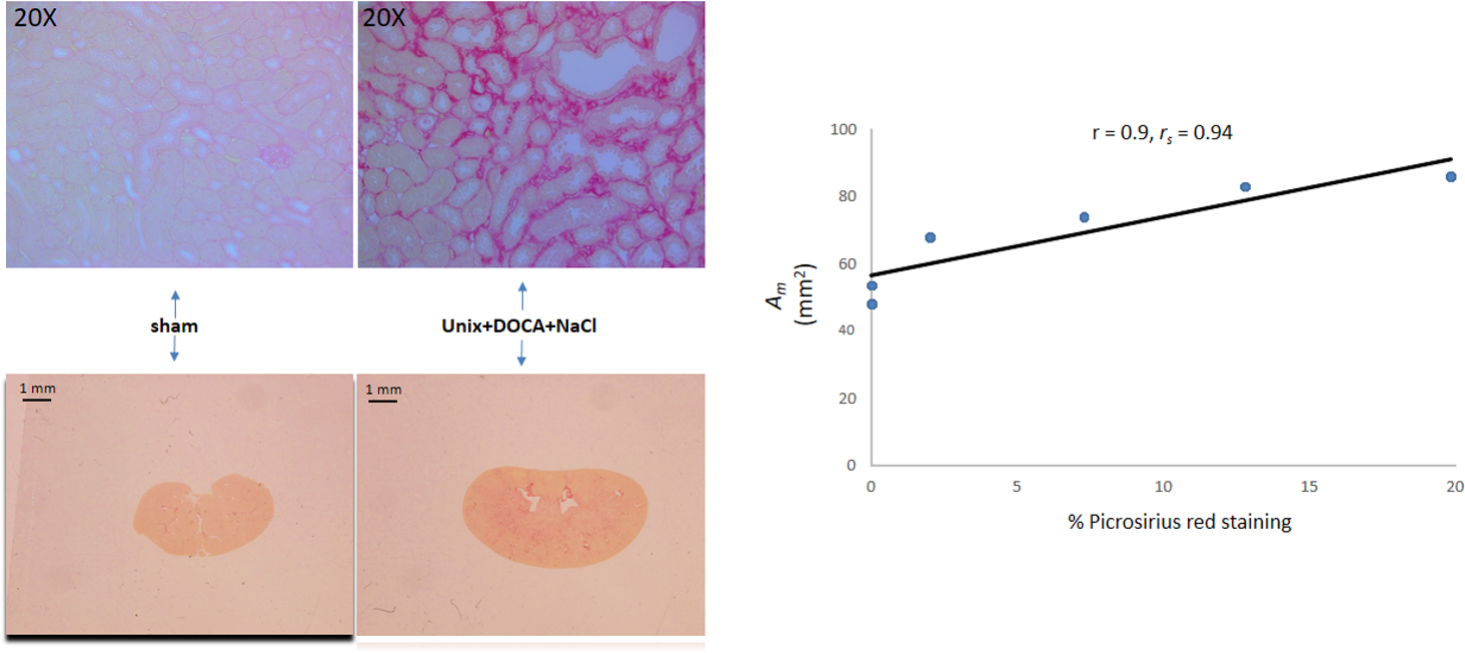
Tubulointerstitial collagen and renal parenchymal area. Picrosirius red stained coronal section (20X) from representative sham and uninephrectomized mouse administered DOCA and NaCl showing rich staining in the renal interstitium. Renal parenchymal area was much larger in the latter cohort. There was a significant correlation between the measured renal parenchymal area (*A*_*m*_) and the extent of tubulointerstitial collagen deposition.

Compared with the sham cohort, Picrosirius red staining was evident within the tubulointerstitium from the uninephrectomy+DOCA+NaCl cohort (Fig 8). Kidneys with increased tubulointerstitial Picrosirius red staining had a larger parenchymal area with a very high correlation between these two variables There is a very high correlation (Fig 3) between these two variables (r = 0.9, p < 0.05; *r*_*s*_ = 0.94, p < 0.01) [16].

## Discussion

In a model of murine uniphrectomy+DOCA+NaCl-induced CKD,_wee report that a) renal fibrosis is evidenced by increased total kidney collagen content or increased tubulointerstitial collagen, b) kidney collagen is directly proportional to renal parenchymal area and c) a new technique involving just two linear axial measurements allows for calculation of renal parenchymal area. Since the calculated parenchymal area is in excellent agreement with the measured or true renal parenchymal area, just two axial measurements informs kidney collagen content and the the extent of renal fibrosis.

Management of the CKD patient continues to present a challenge for the nephrologist given that functional changes are long preceded by extracellular matrix accumulation within the renal interstitium, and given that highly invasive and painful tissue biopsy remains the mainstay for determining the extent of scarring [5]. Furthermore, many patients at highest risk for CKD, including those with coagulation disorders and uncontrolled hypertension, are often not candidates for biopsy [17]. A noninvasive method to capture and track kidney collagen content can obviate the need for repeated biopsies. Intriguingly parenchymal echogenicity and abnormalities in renal size are often evident in CKD [6–8]. While measurement of renal dimensions and calculation of renal volume based on the ellipsoid formula is now standard practice [10], there are little, if any, data translating this information to kidney collagen content. Furthermore, a number of clinical studies [9–11] has shown that use of this formula consistently underestimates kidney volume reported at autopsy.

In the present study, we investigated the renal parenchymal area and corresponding kidney collagen content from healthy mice and DOCA-salt-uninephectomized mice, a standard and well-characterized model of murine CKD [12–13]. A salient feature of this model is that, unlike the subtotal nephrectomy model, the kidney is not surgically perturbed or partially ablated therefore lending itself to reliable measurements of length and width.

Consistent with the afore-referenced reports, entering the standard axial (length and width) measurements into an elliptical formula underestimated renal parenchymal area. By contrast, use of a minor axis measurement that includes the renal pelvis in the elliptical formula yields a parenchymal area that is remarkably consistent with the measured area.

The other hallmark finding of this study was that at least in this model, renal parenchymal area informs kidney collagen content, which is a surrogate for kidney fibrosis. In our sample set, comprising healthy and diseased kidneys, tissue collagen spanned a 4-fold dynamic range with kidneys within a group also exhibiting a range of total collagen. Over this dynamic range, renal parenchymal area tracked tissue collagen evidenced by very similar Pearson product moment and Spearman Rho values, with the latter speaking not only to the strength but also the direction of this relation. Importantly, both the measured parenchymal area, and the parenchymal area calculated using an extended minor axis, returned similar r and *r*_*s*_ values vis. a vis. tissue collagen content. This finding is of translational interest in that, as described by Eq 5, kidney collagen content can now be computed from just two axial measurements across the kidney viz. the major axis and a minor axis that extends into the renal pelvis. This finding was mirrored using the Picrosirius staining technique which identifies both the extent and pattern of fibrosis (14). Compared with kidneys from the sham cohort, kidneys from the uniphrectomy+DOCA+NaCl cohort exhibited rich staining within the renal interstitium. Consistent with the findings from the biochemical assay, renal parenchymal area increased with the extent of tubulointersititial fibrillar collagen deposition. Unlike tissue hydroxyproline measurements, tissue Picrosirius red staining for collagen is semiquantitative in nature and an attempt to quantify the afore-referenced relation was not attempted.

There are certain limitations to our findings. The present study sampled kidneys healthy and diseased mice at a single timepoint of 8 weeks. It remains to be determined whether our findings hold true in murine kidneys of different ages, in other models of CKD such as diabetic nephropathy or in kidneys from higher species including humans. The effects of reverse renal remodeling and potential hysteresis in response to interstitial tissue catabolism on renal parenchymal area remain to be determined. Finally, our findings were drawn from H&E-stained coronal renal slices and may not fully translate to findings drawn using non-invasive imaging modalities such as ultrasound.

These limitations notwithstanding, our results form the foundation for developing a calculator for fibrosis from measurements made during noninvasive renal imaging. Such a calculator will find clinical use along the lines of other existing calculators for renal and liver diseases such as the modified diet in renal disease (MDRD), CKD-epidemiology collaboration (CKD-EPI), FIB-4 and aspartate aminotransferase-to-platelet ratio index (APRI) calculators [18–20]. Such a calculator will not only represent a patient-friendly and relatively inexpensive method to track disease progress and aid in the management of this population but can also potentially be used in clinical trials of drugs that work by reducing the deposition of tissue collagen.

## Supporting information

S1 File Percent Picrosirius red staining, a measure of renal interstitial collagen accumulation, and parenchymal area for the corresponding kidney are listed. (XLSX)
